# Metastatic Cutaneous Melanoma in a White African Lioness (*Panthera leo*)

**DOI:** 10.3390/vetsci8080154

**Published:** 2021-08-01

**Authors:** Louise van der Weyden, Peter Caldwell, Liesl van Rooyen, Emily P. Mitchell, Nicolize O’Dell

**Affiliations:** 1 Wellcome Sanger Institute, Wellcome Genome Campus, Hinxton, Cambridge CB10 1SA, UK; lvdw@sanger.ac.uk; 2 Old Chapel Veterinary Clinic, Villieria, Pretoria 0186, South Africa; peter@oldchapelvet.co.za; 3 IDEXX Laboratories, Barbeque Downs, Johannesburg 1685, South Africa; Liesl-VanRooyen@idexx.com; 4 Department of Paraclinical Sciences, Faculty of Veterinary Science, University of Pretoria, Onderstepoort 0110, South Africa; emily.mitchell@up.ac.za; 5 Centre for Veterinary Wildlife Studies, Faculty of Veterinary Sciences, University of Pretoria, Onderstepoort 0110, South Africa

**Keywords:** lion, melanoma, cutaneous, skin, metastasis, PNL-2

## Abstract

Malignant melanomas tend to be locally destructive, aggressive tumours commonly associated with recurrence and/or metastasis. In this report, a 13-year-old captive white African lioness (*Panthera leo*), with a recent history of intermittent bouts of lethargy and inappetence, presented with a distended abdomen (due to ascites) and a small, round crusty lesion on the ear. An abdominal ultrasound showed the presence of masses on the liver and an exploratory laparotomy revealed multiple pale lesions on the liver and omentum. Histopathology revealed sheets of pleomorphic neoplastic cells compressing the non-neoplastic liver tissue. Similar neoplastic cells had multifocally expanded and effaced omentum adipose tissue, as well as formed a well-circumscribed mass in the ear sample, extending from close to the epidermis to the lateral and deep margins of the section. All three tissue samples had a high mitotic index (15 per 10 HPF), and critically, in the ear sample, there were rafts of neoplastic cells in the lymphatics, indicating lymphovascular invasion. Immunohistochemistry for the melanoma marker, PNL-2, showed strong positivity in all three tissue samples. Thus, the diagnosis was of malignant melanoma with metastasis to the liver and omentum. This is the first report of metastatic cutaneous melanoma in a lion.

## 1. Introduction

Melanomas are neoplasms of neuroectodermal origin that arise from melanocytes and typically occur in the skin (cutaneous melanoma), the digits (digital melanoma), the oral cavity (oral melanoma), and the eyes (ocular melanoma). Melanoma has been reported in most domestic animal species and a number of wildlife species, although it is most commonly found in dogs, horses, and pigs [1]. Melanoma is regarded as uncommon in domesticated cats, with one survey of 3145 feline necropsies identifying only 4 cases [2]. The relative tissue site prevalence for melanoma in cats varies between different studies, with some finding ocular melanoma (intraocular and palpebral) more common than dermal or oral melanoma [3] and others finding cutaneous melanoma more common than intraocular melanoma [2]. Of the non-ocular melanomas, a retrospective study of 23 cases found that the most common locations were the nose, digit, and pinna [4]. Melanomas are rare in non-domesticated felids, with only three reports to date [5,6,7], and only two involving lions: in the eye of a captive African lioness and on the maxillary lip of a captive African lion. The 19-year-old lioness with intraocular melanoma had concurrent mammary mucinous carcinoma and both tumours had widely metastasized upon presentation, so the lioness was euthanized two months later due to her deteriorating condition [5]. The 13-year-old lion with oral melanoma of the inner lip was treated with hypofractionated radiation and immunotherapy (Oncept) to shrink the tumour by 50%, followed by surgical excision. Six months after diagnosis, there were no signs of clinical disease or evidence of metastasis [6]. An aggressive cutaneous melanoma in a non-domesticated felid was recently reported in a 15-year-old captive Siberian tiger (*Panthera tigris altaica*), which developed an ulcerated black nodule caudally to the right ear [7]. The present report describes a cutaneous malignant melanoma on the ear of a white African lioness that was diagnosed due to its metastasis to the liver and omentum, resulting in the development of ascites and necessitating euthanasia due to her deteriorating clinical condition. Metastatic cutaneous melanoma has not previously been reported in a lion.

## 2. Case Presentation

**Figure 1:**
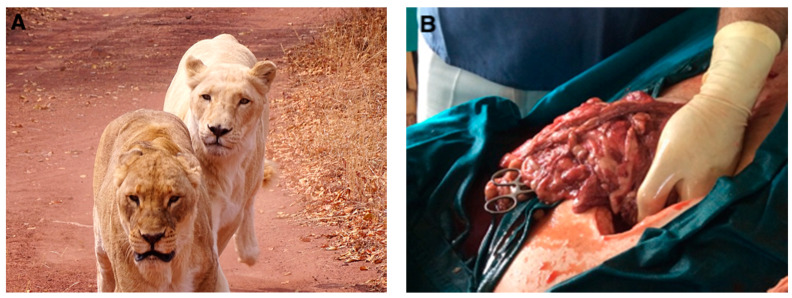
The white lioness with cutaneous melanoma. (**A**) The affected white-coated lioness (**right**), with a healthy normal-coated lioness (**left**). (**B**) Macroscopic photo of the omentum during the exploratory laparotomy.

A 13-year-old intact white African lioness from the Kevin Richardson Wildlife Sanctuary in Pretoria, South Africa (Figure 1A), with a 3–5 day history of intermittent bouts of lethargy and inappetence, presented with a mildly distended abdomen and symptoms of pain, weakness, weight loss, and depression. The sanctuary owner reported the lioness initially had a swollen right ear, but it had since recovered and only a small, round, crusty, black lesion in the right ear remained (5 mm × 5 mm, at the margin of the external ear canal). The lioness was immobilized by intramuscular darting with Medetomidine (20 mLs, 20 mg/mL vial; Kyron Prescriptions, Benrose, South Africa) and Zoletil 100^®^ (active: tiletamine 250 mg, zolazepam 250 mg; Virbac RSA, Centurion, South Africa) to allow for a full clinical examination and blood collection. When compared to internally-derived and laboratory-specific reference intervals for a lion, the complete blood count (Table S1, IDEXX Procyte Dx; IDEXX Laboratories (Pty) Ltd., Westbrook, ME, USA) revealed a mild to moderate microcytic normocytic anaemia without evidence of regeneration: red blood cell count 4.63 × 10^12^/L (RI 5.4–10.32), Hct 0.185 L/L (RI 0.28–0.52), Hgb 67 g/L (RI 90–170), MCV 40 fL (RI 41.9–56.76) and MCHC 362 g/L (RI 281–389). The anaemia was interpreted as being most consistent with anaemia of inflammatory disease. The leukocytosis present was due to a moderate neutrophilia and monocytosis: white blood cell count 24.92 × 10^9^/L (RI 6–16.2), absolute neutrophil count 21.05 × 10^9^/L (RI 3.6–13.69), absolute monocyte count 1.56 × 10^9^/L (RI 0.08–0.63). These findings were interpreted as being the most consistent with an inflammatory leukogram. The clinical chemistry profile (Table S2, IDEXX Catalyst One; IDEXX Laboratories (Pty) Ltd., Westbrook, ME, USA) revealed mild hyperproteinaemia due to hyperglobulinaemia, suggestive of either dehydration or the increased synthesis of acute-phase proteins and/or immunoglobulins: total protein 90 g/L (RI 62–87); globulins 62 g/L (RI 22–59). Additionally, the serum urea concentration was mildly decreased and a mild hyperbilirubinaemia was present; the combination thereof suggested altered liver function: urea 6 mmol/L (RI 6.9–15.9) and total bilirubin 17 μmol/L (RI < 5.4). A marginal hypokalaemia was also present, which was most likely a result of decreased intake: 3.6 mmol/L (RI 3.7–4.9). In addition to the aforementioned in-house diagnostics, serum was submitted to IDEXX Reference Laboratories, Johannesburg, South Africa, for a serological viral screening profile. The results did not suggest any recent direct exposure to any of the common feline viral infections (Table S3).

Since the blood results were suspicious for infection (as peritonitis and pyometra are common in older lions), inflammation (such as osteoarthritis), and liver disease, two days later the lioness was started on an oral course of Clavumox^®^ (1 g tablets, 2½ tablets, twice per day for 10 days; Cipla Medpro, Cape Town, South Africa); Tolfedine^®^ (60 mg tablets, 2 tablets once per day for 4 days; Afrivet, Pretoria, South Africa); Tramahexal (100 mg tablets, 1½ tablets once per day for 5 days; Salutas Pharma GmbH, Barleben, Germany); Solal SAMe, (400 mg tablets, 1 tablet once per day for 14 days; Ascendis Supply Chain, Gauteng, South Africa).

At the same time, an exploratory laparotomy was planned to rule out pyometra and determine the cause of the distended abdomen. Twelve days later, the lioness was immobilized by intramuscular darting with Medetomidine and Anaket-V^®^ (active: ketamine hydrochloride, 10 mLs, 100 mg/mL vial; Bayer, Isando, South Africa) and placed on an intravenous electrolyte solution (Ringers Lactate; Fresenius Kabi, Bad Homburg, Germany). She was then intubated and maintained under anaesthesia using isofluorane. The abdominal area was shaved and prepared for surgery. During the exploratory laparotomy, all abdominal organs were evaluated. The liver and omentum contained multiple widespread, pale, nodular masses of various sizes, thought to be neoplasia or granulomatous inflammation (Figure 1B). In addition, the abdominal cavity was filled with serosanguineous fluid. Biopsies of the liver, omentum, and the affected piece of the ear were placed in a 10% neutral-buffered formalin for histopathological analysis at the Faculty of Veterinary Science, University of Pretoria, South Africa. Samples of the abdominal ascitic fluid were taken for cytological analysis at IDEXX Laboratories (IDEXX Reference Laboratories, Johannesburg, South Africa). Given the severity of the lioness’ clinical condition, the animal was immediately euthanized. The owner declined a post-mortem examination.

The abdominal fluid sample was processed in accordance with laboratory standard operating procedures. Fluid analysis revealed a nucleated cell count (NCC) of 24,000/μL, and a total protein concentration of 42 g/L was obtained by means of refractometry. For the preparation of the cytological smears, 200 μL of fluid were centrifuged using a cytocentrifuge and stained with a rapid Romanowsky stain. The abdominal ascitic fluid sample showed high numbers of highly anaplastic round to oval cells arranged individually and in clusters. The cells displayed severe anisocytosis and had variably well-defined cell borders. The cytoplasm was medium to deeply basophilic and vacuolated with variable nuclear: cytoplasm ratios. The nuclei were oval to reniform, eccentrically placed, and showed severe anisokaryosis, coarsely stippled chromatin, large prominent nucleoli, nuclear moulding, irregular contours, multinucleated cells, and atypical mitoses. Moderate numbers of vacuolated, pigment-laden macrophages and low numbers of peripheral blood leukocytes and plasma cells were present and no infectious agents were seen. The diagnosis was a neoplastic effusion, with the cytologic features the most consistent with carcinoma. Considering the history of the lioness, it was felt that the most obvious site of origin would be the liver.

**Figure 2:**
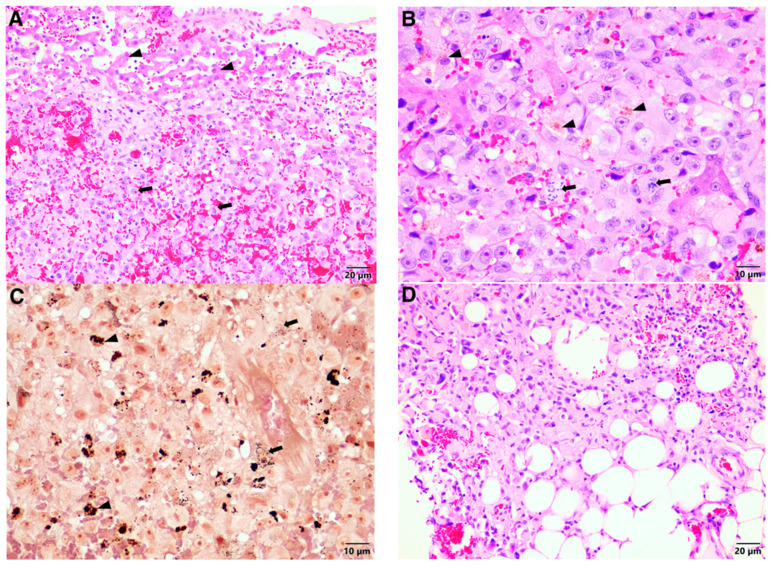
Malignant melanoma, lion: histopathology of the liver and omentum. (**A**) Poorly defined proliferation of neoplastic cells (*arrows*), compressing a small area of non-neoplastic hepatocytes (*arrowheads*). (**B**) Pleomorphic round to oval to polygonal neoplastic cells with small to large amounts of pale eosinophilic finely vacuolar cytoplasm. Mitoses were present in all high power fields (*arrows*) and brown pigment-laden cells were common (*arrowheads*). (**C**) The pigment within the neoplastic cells stained typical for melanin using Fontana-Masson special stain. The melanin pigment varied from finely granular (*arrows*) to coarsely granular (*arrowheads*). (**D**) Proliferating neoplastic cells within the adipose tissue of the omentum.

The formalin-fixed tissues were embedded in paraffin wax, sectioned, and stained with haematoxylin and eosin. An irregularly nodular section of the liver (4 × 2.5 × 2 cm) contained neoplastic cells arranged in poorly defined trabeculae and sheets extending from the capsular surface to the margins of the tissue that compressed the small areas of non-neoplastic liver tissue (Figure 2A).

**Figure 3:**
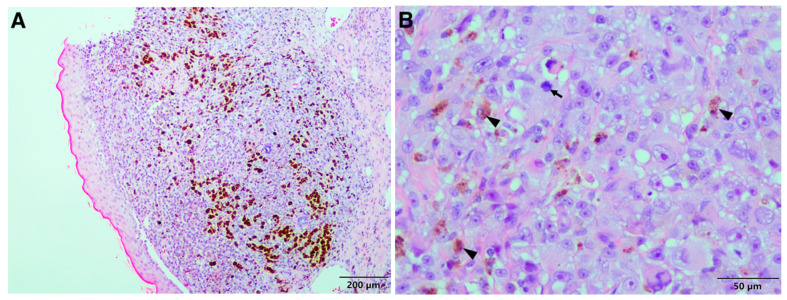
Malignant melanoma, lion: histopathology of the skin mass on the ear. (**A**) Proliferation of neoplastic cells in the dermis similar to those found in the liver and omentum. The neoplastic cells extend close to the epidermis and brown pigment is clearly visible. (**B**) Neoplastic cells similar to those found in the liver and omentum within the dermis containing brown cytoplasmic pigment (*arrowheads*). Note the mitotic figure (*arrow*).

The neoplastic cells were pleomorphic, round to oval to polygonal in shape, with large to small amounts of pale eosinophilic finely vacuolar cytoplasm. They had large, round, often indented (and occasionally multiple), often vesicular, nuclei, and large central amphophilic nucleoli. The mitotic index was 16 per 10 HPF. A few neutrophils and lymphocytes had infiltrated the neoplasm. Individual cell necrosis and brown pigment-laden cells were common (Figure 2B), as were large areas of haemorrhage and apparent telangiectasia. The brown pigment within the neoplastic cells stained typical for melanin using the Fontana-Masson special stain (Figure 2C). A section of omentum (13 × 3 × 1 cm) contained scattered proliferating nodules and an irregular soft brown discolouration at one pole. Similar neoplastic cells to those seen in the liver sample were found multifocally expanding and effacing the adipose tissue, mixed with variable numbers of lymphocytes and plasma cells (Figure 2D). The mitotic rate was similar to the liver sample. The sample from the ear consisted of a grey-black nodular section of tissue attached to a strip of normal-looking skin (2 × 1 × 0.5 cm). Similar neoplastic cells to those seen in the liver and omentum formed a well-circumscribed compact mass extending from close to the epidermis to the lateral and deep margins of the section (Figure 3A). The mitotic index was 15 per 10 HPF. Numerous neoplastic and phagocytic cells contained brown pigment in the cytoplasm (Figure 3B), and rafts of neoplastic cells were seen in the lymphatics.

**Figure 4:**
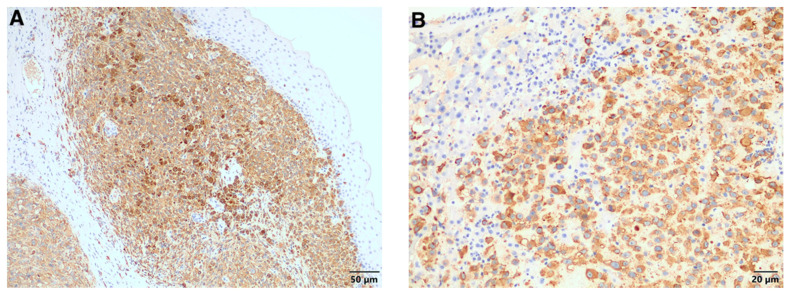

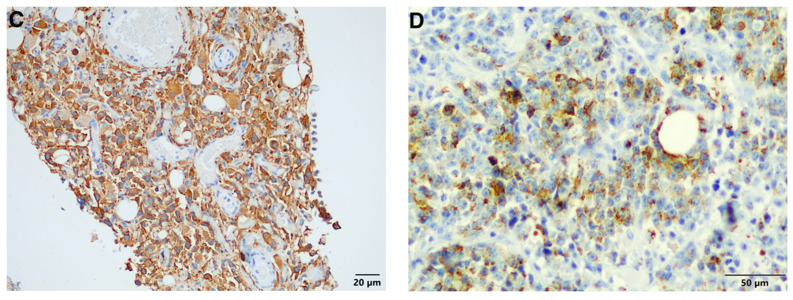
Positive PNL-2-specific labelling of the neoplastic cells in the (**A**) skin, (**B**) liver, and (**C**) omentum. (**D**) Positive PNL-2 control.

Based on the diagnosis of melanoma in the ear, a presumptive diagnosis of metastatic melanoma was made for the liver and omental masses. To further characterize the neoplastic cells, 4 μm serial sections of the paraffin blocks were cut and used for indirect immunohistochemistry. Antigen retrieval was performed by heat treatment in 10 mM citrate buffer, pH 6.0 for 15 min. Thereafter, sections were treated for 15 min with 3% hydrogen peroxide in methanol (to block endogenous peroxidases). They were then incubated for 2 h at room temperature with a mouse monoclonal PNL-2 antibody (1:300 dilution; Santa Cruz Biotechnology, Dallas, TX, USA) as a primary antibody, which is a marker that demonstrates high sensitivity for melanoma, with superior specificity to S-100 [8] and has been previously shown to be a sensitive marker for feline non-ocular melanocytic neoplasms [9]. Slides were then treated with a polymer detection kit as per the manufacturer’s instructions (Super Sensitive™ Polymer-HRP; Biogenex, Fremont, CA, USA). A Vector^®^ NovaRED^®^ Substrate Peroxidase Kit was used as a chromogen (Vector Laboratories, Burlingame, CA, USA), and the slides were then counter-stained using Mayer’s haematoxylin, dehydrated, and mounted with Entellan for evaluation via light microscopy. Sections of confirmed canine melanoma tissue were used as a positive control and negative internal dermal collagen was used for negative control purposes. The cutaneous mass (Figure 4A), the masses in the liver (Figure 4B), and those in the omentum (Figure 4C) stained strongly positive with PNL-2 compared to the positive control (Figure 4D). The location of the positive staining was predominantly cytoplasmic and constituted approximately 100% of the neoplastic population in all locations. Thus, a diagnosis of metastatic malignant cutaneous melanoma was made.

## 3. Discussion

The neoplasia, in this case, was a highly malignant melanoma on the pinna which metastasized to the liver and omentum. Metastasis to other locations, such as the lymph nodes and lungs, may have taken place; however, the owner refused a post-mortem examination, so it was not possible to assess this. Malignant melanoma is a neoplasm commonly associated with an unfavourable prognosis in both humans and animals due to its propensity to metastasize. In domestic cats, intraocular melanoma and melanomas arising in the oral cavity, lips, and nose are associated with higher metastatic potential and/or decreased survival time, relative to cutaneous melanomas (of the skin or digit) [3,9]. However, in the present report of cutaneous melanoma, a high mitotic index, as well as a high degree of cellular and nuclear pleomorphism and metastasis were observed, similar to that reported for metastatic cutaneous melanoma in a Siberian tiger (*Panthera tigris altaica*) [7]. Interestingly, a mitotic index has been demonstrated to be a predictive prognostic factor for melanocytic neoplasia in domestic cats and dogs [10].

In humans, melanomas are classified according to sun exposure, specifically melanoma arising in chronically sun-exposed skin, intermittently sun-exposed skin, or sun-protected skin [11]. Ultraviolet (UV) light is the major cause of melanoma development in sun-exposed skin in humans [12]. However, whilst UV exposure is responsible for causing some skin cancers in cats, such as cutaneous squamous cell carcinoma, it has not yet been identified as a risk factor for cutaneous melanoma. Yet, the lioness in this report was of white coat colour; white lions are a rare colour variant of the African lion (*Panthera leo*), which is typically tawny in colour. The white coat colour is due to leucism (partial loss of pigmentation) resulting from a double recessive allele [13], rather than due to albinism (absence of melanin) [14], and it is tempting to speculate that it may predispose them to the development of cutaneous melanoma. Evidence of a link between hair colour and tumour pathogenesis in animals has already been demonstrated. For example, white-haired cats have ~13 times the risk of developing cutaneous and oral squamous cell carcinoma than cats of other hair colours [15]. In addition, cutaneous melanoma had not been reported in wildebeests (*Connochaetes taurinus*) until a recent study, which found four cases in golden and king wildebeest colour variants, suggesting that the paler coat colour potentially predisposes them to the development of melanoma [16].

Non-ocular melanoma is a disease of older domesticated cats [3,9,17]. Cats with non-ocular melanoma are most commonly treated with complete surgical removal of the tumour (depending upon its size and location), although radiation therapy (for non-surgical tumour control or after surgery if complete excision was not achieved) and chemotherapy (together with surgery or radiotherapy) have also been used [17]. There is also a report of successful treatment of a 13-year-old lion with oral melanoma, which was achieved with hypofractionated radiation therapy to shrink the mass, followed by surgical excision, and there were no clinical signs of disease or evidence of metastasis at six months after diagnosis [6]. Interestingly, a study of tissue margins in samples of non-ocular melanoma that had been surgically removed found that the margins were only histologically clean in 14 out of 19 cases (74%), and cats with a clean surgical margin had a significantly better outcome than those with infiltrated margins (*p* < 0.001) [17].

In general, malignant melanomas, especially of the skin, are easily diagnosed, however, those presenting as non-cutaneous primaries or as a metastatic disease can closely mimic other tumour types [18]. A retrospective study of 141 feline non-ocular melanomas found there was a wide variation in their histologic appearance [9]. Indeed, the neoplastic cells, in this case, were pleomorphic in appearance, although similar between the three different tissues. Critically, it was the submission of an unrelated skin lesion that led to the diagnosis of melanoma. In this case, the use of immunohistochemistry with an anti-PNL-2 antibody, confirmed the diagnosis of metastatic melanoma in the liver/omentum samples, as PNL-2 is a sensitive marker to confirm the melanocytic origin in cats [8].

## 4. Conclusions

In conclusion, cutaneous melanoma, although rarely reported in non-domesticated felids, should be considered as a differential diagnosis for any skin mass. Early detection is essential as it seems that cutaneous melanoma can be a very aggressive tumour with a high propensity to metastasize, yet if caught early, disease-free survival is possible. It is hoped that further investigations of lions, both in wildlife reserves and zoological facilities, will add to a better knowledge and understanding of melanomas in this species, to promote their early detection.

## Data Availability

All the data reported by this case study is contained within the article or supplementary material.

## Supplementary Materials

The following are available online at https://www.mdpi.com/article/10.3390/vetsci8080154/s1, Table S1: Diagnostic haematology laboratory report for the lioness, Table S2: Diagnostic serum chemistry laboratory report for the lioness, Table S3: Diagnostic viral screen laboratory report for the lioness.
