# Supplementary material for: Metastatic Cutaneous Melanoma in a White African Lioness (Panthera leo)
Source: Vet Sci. 2021 Aug 1;8(8):154. doi: 10.3390/vetsci8080154 (PMC8402825; doi:10.3390/vetsci8080154)
Supplement: Supplementary file 1 [file vetsci-08-00154-s001.zip › vetsci-1303259-updated supplement.pdf]

cancers

MDPI

Supplementary Materials

Metastatic Cutaneous Melanoma in a White African Lioness (*Panthera leo*)

Louise van der Weyden, Peter Caldwell, Liesl van Rooyen, Emily P. Mitchell and Nicolize O'Dell

Table S1. Diagnostic haematology laboratory report for the lioness. The laboratory-specific reference intervals in use were derived from statistical analysis of results generated from lion samples submitted to IDEXX Laboratories (Pty) Ltd (South Africa).

Test	Result	Reference interval
RBC (× 10^{12}/L)	4.63	5.4 – 10.32
Haematocrit (L/L)	0.185	0.28 – 0.52
Haemoglobin (g/L)	67	90 – 170
MCV (fL)	40	41.1 – 56.6
MCH (pg)	14.5	N/A
MCHC (g/L)	362	281 – 389
RDW (%)	27.8	N/A
Reticulocytes (× 10^9/L)	44.9	N/A
Reticulocyte haemoglobin	15.3	N/A
WBC (× 10^9/L)	24.92	6.1 – 16.2
Neutrophils (× 10^9/L)	21.05	3.6 – 13.69
Lymphocytes (× 10^9/L)	2.28	0.4 – 2.6
Monocytes (× 10^9/L)	1.56	0.08 – 0.63
Eosinophils (× 10^9/L)	0.00	0.08 – 0.56
Basophils (× 10^9/L)	0.03	0 – 0.018
Platelets (× 10^9/L)	359	68 – 523
MPV (fL)	16.4	N/A
Plateletcrit (%)	0.59	N/A

Table S2. Diagnostic serum chemistry laboratory report for the lioness. The laboratory-specific reference intervals in use were derived from statistical analysis of previous results generated from lion samples submitted to IDEXX Laboratories (Pty) Ltd (South Africa).

Test	Result	Reference interval
Glucose (mmol/L)	5.34	4.1 – 9.35
IDEXX SDMA (µg/dL)	18	N/A
Creatinine (µmol/L)	189	95 – 352
Urea (mmol/L)	6.0	6.9 –15.9
Phosphorus (mmol/L)	1.81	1.27 – 2.19
Calcium (mmol/L)	2.20	2.16 – 2.73
Sodium (mmol/L)	154	142 – 160
Potassium (mmol/L)	3.6	3.7 – 4.9
Chloride (mmol/L)	106	109 – 129
Total Protein (g/L)	90	62 – 87
Albumin (g/L)	29	22 – 44
Globulin (g/L)	62	22 – 59
ALT (U/L)	42	21 – 72
ALP (U/L)	<10	4 – 33

GGT (U/L)	0	0 – 4
Bilirubin – Total (µmol/L)	17	0 – 5.4
Cholesterol (mmol/L)	2.67	2.3 – 6
Triglyceride (mmol/L)	0.48	0.17 – 0.64
Amylase (U/L)	>2500	N/A
Lipase (U/L)	109	N/A

Table S3. Diagnostic viral screen laboratory report for the lioness. The assays for Calici Virus, Herpes Virus, Feline Corona Virus and Panleukopaenia Virus are quantitative fluorescent antibody tests, which indicate antibody status as a result of direct or indirect exposure to disease, or vaccination status. In this case, a dilution of 1:20 is very low, indicating a low antibody titre, which makes active infection less likely; thus it was not deemed necessary to do further testing such as PCR or antigen testing. The FIV antibody test as part of the IDEXX SNAP FIV/FeLV Combo test is a qualitative rapid screening test which detects the presence of serum antibodies. The viral testing for FELV detects the presence of viral antigen in the serum.

Test	Result
Feline Calici Virus Antibody (Dilution 1:20)	Positive
Feline Herpes Virus Antibody (Dilution 1:20)	Positive
Feline Panleukopaenia Virus Antibody (Dilution 1:20)	Positive
Feline Corona Virus Antibody	Negative
Feline Immunodeficiency Virus (FIV)	Negative
Feline Leukaemia Virus (FELV)	Negative
